# Evaluation of Preharvest Melatonin on Soft Rot and Quality of Kiwifruit Based on Principal Component Analysis

**DOI:** 10.3390/foods12071414

**Published:** 2023-03-27

**Authors:** Junsen Peng, Shouliang Zhu, Xin Lin, Xuan Wan, Qin Zhang, Alagie Njie, Dengcan Luo, Youhua Long, Rong Fan, Xiaoqing Dong

**Affiliations:** 1 Fruit Crops Center of Guizhou Engineering Research, College of Agricultural, Guizhou University, Guiyang 550025, China; pjs19980416@163.com (J.P.); a.njie@utg.edu.gm (A.N.);; 2 Guizhou Workstation for Fruit and Vegetables, Guiyang 550025, China; shouliang2006@163.com; 3 Engineering and Technology Research Center of Kiwifruit, Guizhou University, Guiyang 550025, China; gzlyh126@126.com (Y.L.);

**Keywords:** melatonin, preharvest, soft rot, quality, kiwifruits

## Abstract

*Botryosphaeria dothidea* is the source of the deadly kiwifruit disease known as soft rot. In order to explore the role of melatonin in regulating the postharvest quality and disease resistance of kiwifruit at different growth and development stages, in this study, we applied melatonin at different concentrations to kiwifruit at the young fruit, expansion, and late expansion stages to assess its effect on fruit resistance to *B. dothidea*, minimize soft rot, and maintain postharvest fruit quality. The results showed that melatonin significantly suppressed the mycelial growth of *B. dothidea*, with 1.0 mmol/L melatonin inhibiting it by up to 50%. However, 0.1–0.3 mmol/L melatonin had the best control over soft rot. Furthermore, spraying MT during kiwifruit growth can successfully increase fruit weight; preserve postharvest fruit firmness; reduce respiration intensity in the early stages of storage; delay the rise in soluble solids, while maintaining a high titratable acid content to ensure suitable solid acid ratio; increase total phenol, flavonoid, chlorophyll, carotenoid, and ascorbic acid contents; and delay the rise in soluble sugar contents in the late stages of storage. These results have a positive effect on maintaining the nutritional composition of kiwifruit. However, the effects on weight loss, dry matter content, and soluble protein content were not significant. In addition, the results of the principal component analysis demonstrated that 0.3 mmol/L MT increased kiwifruit’s resistance to soft rot while preserving postharvest fruit quality.

## 1. Introduction

One of the four most popular domesticated fruit trees in the 20th century, kiwifruit (*Actinidia deliciasa*) is a deciduous vine fruit tree that produces fruit with a high nutritional content and is adored by consumers for its sweetness and taste [1,2]. Unfortunately, kiwifruits are climacteric fruits that are susceptible to mildew and postharvest deterioration. The main factor causing kiwifruit rot during storage is fungus, and one of the major diseases affecting kiwifruit is soft rot, caused by *Botryosphaeria dothidea* [3,4]. Chemical fungicides are a standard means of preventing soft rot; however, when misused, they can render the infections more resistant, seriously pollute the environment, and harm human health [5,6,7].

Natural fungicides from plants have drawn more interest as low-toxicity, environmentally acceptable substances that increase host resistance [8,9]. Melatonin (MT) is a tiny molecular indole chemical widely distributed throughout animals, plants, and microbes. It has been conserved throughout biological evolution. Numerous studies have documented how melatonin regulates plant development, abiotic stress, and postharvest preservation [10,11,12]. MT has been actively use in recent years in promoting fruit disease resistance. According to Xiang et al. [13], MT treatment dramatically reduced the lesion diameter spread on pears infected with *Alternaria alternata.* Fan et al. [14] discovered that MT preserved guava fruit quality and increased resistance to anthracnose by lowering the levels of hydrogen peroxide (H_2_O_2_) and malondialdehyde (MDA). Total immersion of litchi in MT solution caused resistance by modifying phenylpropane and energy metabolism [15]. Similar findings have been confirmed for apples and grapes [16,17]. Previous studies discovered that the soft rot pathogen infects kiwifruit during the fruit’s growth and causes subsequent pathological changes [18]; a single postharvest treatment did not successfully prevent the occurrence of soft rot. Therefore, preharvest control is crucial for preventing soft rot of kiwifruit, and researchers are interested in a preharvest application to prevent postharvest disease [19,20]. For instance, Li et al. [21] discovered in a field study that treating kiwifruit with 0.50 mmol/L MeJA one month preharvest successfully avoided and controlled soft rot by up to 77.70%, effectively improving fruit appearance and quality. According to Zhang et al. [22], preharvest spraying of chitosan composite film (CCF) significantly raised the concentration of resistance compounds and activated defense enzyme activity while dramatically reducing the incidence of soft rot.

Preharvest MT treatment promotes normal plant growth and enhances fruit yield and quality against abiotic stress. In addition, MT increased the soluble sugar (SS), β-carotene, vitamin C (Vc) content, and total soluble solid (TSS) contents of tomato fruits during salt stress [23]. Furthermore, spraying MT at late tomato expansion during biotic stress reduced tomato gray mold and the naturally occurring decay of tomato fruit during storage. Additional transcriptome analysis revealed that MT upregulated the expression of calcium-dependent protein kinase (SlCDPK) and respiratory burst oxidase homologs (SlRBOHs) associated with reactive oxygen species burst, increased salicylic acid (SA) and lignin contents, and increased the expression of most of the SA signaling pathway and phenylprop [24,25]. Currently, MT is primarily used in postharvest fruit treatment research; yet, it is unknown whether preharvest MT spraying can enhance kiwifruit fruit quality and disease resistance. It is therefore of utmost importance to investigate how MT affects kiwifruit’s postharvest quality and disease resistance at various growth stages. Additionally, there is a lack of in-depth research on how melatonin coordinates the relationship between fruit quality and resistance.

This study primarily examined the effect of MT preharvest treatment on postharvest quality and disease resistance of ’Guichang’ kiwifruit and determined a rationale for and practical recommendations for using MT in the postharvest management of fruit and vegetable diseases.

## 2. Materials and Methods

### 2.1. Materials

*Botryosphaeria dothidea* was provided by the Department of Plant Protection, College of Agriculture, Guizhou University, which was subculture don potato dextrose agar (PDA) medium. The following compounds were used in the experiment: melatonin, rutin, gallic acid, trichloroacetic acid, red phenanthroline, anthrone, sodium nitrite, aluminum nitrate, Coomassie brilliant blue G-250, sodium hydroxide, and sodium carbonate, which were purchased from Sigma-Aldrich (Shanghai) Trading Co., Ltd. (Shanghai, China). In addition, ethanol, acetone, dimethyl sulfoxide (DMSO), and ethyl acetate were obtained from Tianjin Kemiou Chemical Reagent Co., Ltd. (Tianjin, China). All other chemicals were analytical grade in this study and purchased from Beijing Solar Science & Technology Co., Ltd. (Beijing, China).

### 2.2. In Vitro Melatonin Inhibition Test

In order to screen doses for field research, a pre-experiment was necessary to assess the inhibitory impact of MT on *B. dothidea*. The procedure was carried out as described by Pan et al. [4]. The methods included the preparation of PDA medium and MT solution at concentrations of 0 (CK), 0.1, 0.3, 0.5, and 1.0 mmol/L (using DMSO as solvent); sterilization (121 °C for 20 min) in an autoclave (Jiangyin Binjiang Medical, Jiangyin, China); and cooling in a super-clean bench (Zhucheng Yu Yang Food Machinery, Shanghai, China). The *B. dothidea* colonies were pierced with a stopper borer (6 mm diameter), positioned in the middle of a medium containing various MT concentrations (containing 0.05% DMSO by volume), and incubated at 28 °C in an incubator (Changzhou Maikenuo, Changzhou, China). Daily observations and data collection were performed during this time. When the control colonies had grown to the full diameter of the petri dish, a Vernier caliper (Airaj Tools, Germany) was used to take measurements. The inhibition rate was estimated using the formula below, with each concentration replicated three times. Inhibitory rate (%) = [(control colony diameter − mycelial disks diameter) − (treatment colony diameter − mycelial disks diameter)]/(control colony diameter − treatment colony diameter) × 100.

### 2.3. Field Experiments

The kiwifruit cultivar *Guichang* was planted in a commercial base in Xiuwen County, Guiyang city, China (27°3′48″ N, 106°26′26″ E). Freshly made 0.1, 0.3, 0.5, and 1.0 mM MT solutions containing 1 mL·L^−1^ Tween were applied to trees, with distilled water containing 1 mL·L^−1^ Tween as the control. A randomized block design was used to arrange the twenty plots. Four kiwifruit trees per plot, one replicate of each tree, and four replicates of each treatment were used. A manual sprayer was used, and two liters of foliar spray was applied to each tree after 6 p.m. during early fruit set, fruit expanding, and late expanding periods. No rainfall was observed 3–4 days after MT spraying.

### 2.4. Sampling and Analysis of Fruit

The fruits with 6.5–7.0% soluble solids were collected 146 days after blossoming in plots of 100 fruits each. Two sets of fruits were separated. Fruit firmness, respiration rate, and internal quality were evaluated using the first group. The second group was used to inoculate *B. dothidea* to observe the diameter of the lesions and the noninoculated fruits in terms of natural incidence. All fruits were placed in perforated cardboard boxes, packed into transparent polyethylene terephthalate (PET) packaging boxes (23 cm × 15 cm × 5 cm), and kept in a warehouse at room temperature between 22 and 25 °C and 70–80% relative humidity. Samples were collected after two days to measure fruit firmness, internal quality, and natural incidence. The pulp samples were quickly frozen with liquid nitrogen and put in an ultra-low-temperature freezer (Aucma, DW-86L630, Qingdao, China) set at −80 °C to determine other indicators later.

### 2.5. Fruit Soft Rot Resistance Test

#### 2.5.1. Effects of Preharvest MT on Postharvest Kiwifruit Inoculated with *B. dothidea*

Seventy-two fruits were randomly selected from the second group from both preharvest MT-treated and control fruits. They were sterilized in 2% (*v*/*v*) sodium hypochlorite for 10 min before being dried by UV light on a super-clean bench. We applied 75% (*v*/*v*) alcohol to the fruit’s equator, and the inoculation site was documented for future use. The mycelial disks were taken from the 7-day *B. dothidea* colony using a stopper borer (6 mm diameter), which was then adhered to a round wound made at the kiwifruit equator using a scalpel, mycelial disks, wet cotton, and parafilm. The lesion diameter of the inoculated fruits was measured daily.

#### 2.5.2. Effects of Preharvest MT on Disease Incidence and Severity of Postharvest Kiwifruit

Preharvest MT treatment and control were set up in 4 boxes containing 160 kiwifruits. The incidence of fruit soft rot was examined two days after the fruit started to soften [22]. The disease severity scale used was as follows: 0, no disease; 1, cumulative lesion diameter less than 1 cm; 2, cumulative lesion diameter of 1–2 cm; 3, cumulative lesion diameter of 2–3 cm; 4, cumulative lesion diameter of 3–4 cm; 5, cumulative lesion diameter of 4–5 cm; 6, cumulative lesion diameter greater than 5 cm.

Disease incidence (%) = Number of diseased fruits/the total Number of survey results × 100(1)

Disease severity index = ∑ (Disease severity value × No. of the fruit within each disease severity value)/(Total No. of fruit × the highest disease severity value) × 100(2)

### 2.6. Effects of Preharvest Melatonin on Quality Parameters of Kiwifruit

With a Vernier caliper (AirajTools, Germany), each fruit’s longitudinal, transverse, and lateral diameters were measured. The fruit shape index was determined using the longitudinal diameter/transverse diameter, and fruit volume was determined using the ellipsoid volume formula to calculate the approximate volume of the kiwifruit. Finally, twenty fruits were measured in each replicate using an electronic balance (Want Balance, Shanghai, China) to weigh a single fruit.

Fruit firmness was measured using a digital readout fruit pressure tester (GY-4, Handpi, China), and 18 fruits were randomly selected from the control and treatment groups. After peeling, r places along the fruit’s equator were chosen, using a 3.5 mm probe and each location was pressed for 5 mm, and the results are expressed in (N).

Using a PAL-BX/ACID1 sugar acidity meter, total soluble solid (TSS), titratable acidity (TA), and solid acid ratio (TSS/TA) were determined (ATAGO, Tokyo, Japan). Then, 18 randomly selected fruits from each treatment group were selected, and the pedicles were chopped into little pieces to determine TSS, TA, and TSS/TA. Finally, the average of each fruit’s two measurements was calculated.

Dry matter determination was carried out using a drying process [22]. Three fruits were chosen randomly, peeled, and sliced into thin pieces measuring approximately 0.5 cm on four sides. The pieces were baked in an aluminum box for 16 h at 70 °C in an oven (Ouwen Oven Manufacturing, Suzhou, China). To determine whether the weight was the same before and after the two weighs, the aluminum box was removed and weighed after drying for an hour and after the completion of the drying process. The outcomes were averaged out. Dry matter (%) = [(*M*2 − *M*0)/(*M*1 − *M*0)] ×100, where *M*0 is the weight of the aluminum box, (g); *M*2 is the weight of the test sample and the aluminum box; *M*1 is the weight of the aluminum box and sample after drying.

Soluble sugar (SS) content was measured by anthrone colorimetry [26]. First, 0.2 g of tissue sample was placed in a test tube, and 10 mL of distilled water was added and sealed with parafilm. After boiling the test tube twice for 30 min, the extract was filtered into a 25 mL volumetric flask. The test tube and residue were repeatedly rinsed, and the volume was fixed to the scale. Next, 0.5 mL of sample extract, 1.5 mL distilled water, 0.5 mL anthrone ethyl acetate reagent, and 5.0 mL concentrated sulfuric acid were added and mixed by vortexing. The test tube was then placed in a pot of boiling water, boiled for 1 min, removed, and allowed to cool to room temperature. The absorbance (OD) value was determined at 630 nm with a UV-5500 UV–Visible spectrophotometer (Shanghai Metash Instruments, Shanghai, China). The results are expressed in mass fraction.

The Coomassie brilliant blue method was used for soluble protein (SP) determination [27]. First, the kiwifruit sample (1 g) was mixed with 5 mL of distilled water, and then the solution mixture was centrifuged at 12,000× *g* for 20 min at 4 °C using a TGL-16M centrifuge (Pingfan Science & Technology, China). Next, the supernatant (1 mL) was added to a test tube with 5 mL of Coomassie brilliant blue G-250 solution. After 2 min of thorough mixing, the absorbance value was measured at 595 nm. The results are expressed as the mass of soluble protein per gram of fruit (mg/g).

Ascorbic acid (AsA) was assessed using the red phenanthroline method [28]. First, 10 g of tissue was ground into a homogenate and transferred to a 100 mL flask, and the volume was adjusted to the scale using the TCA solution. After mixing and extracting for ten minutes, the filtrate was filtered, and we collected the supernatant. Next, 1 mL of the sample extract was added to 1 mL TCA solution, and then 0.5 mL of the 0.4% phosphoric acid-ethanol, 1 mL of the BP-ethanol, and 0.5 mL of the FeCl_3_-ethanol were added and mixed by vortexing. The absorbance of the solution was measured at 534 nm. The results are expressed by the mass of AsA contained in a 100 g sample (mg/100 g).

The extraction to measure carotenoids and chlorophyll was carried out using the method of Zhang et al. [29]. After dark extraction, the kiwifruit sample (1 g) was added to 10 mL of acetone, 95% ethanol (2:1), and a small amount of CaCO_3_ for 7 h. The absorbance at 649, 665, and 470 nm was determined, and the contents of chlorophyll a, chlorophyll b, and carotenoid were calculated using the Arnon formula.

According to the method of Zhang et al. [15], total phenols (TP) and total flavonoids (TF) were determined. First, the kiwifruit sample (2 g) was mixed with 5 mL of precooled 60% ethanol, ground, and homogenized in an ice bath before being transferred to a 10 mL test tube. The supernatant was then collected at (4 °C, 12,000× *g*, 20 min) to produce the phenolic extract, which was then stored at a low temperature for later use. Afterward, 1 mL of the extract solution was mixed with 6 mL of distilled water, 0.5 mL of Folin–Ciocalteu reagent was added, and then rested for 5–8 min before adding 1.5 mL of 20% Na_2_CO_3_ solution and diluting with water to 10 mL. The reaction solution was kept at 25 °C for 1 h, and the absorbance value was measured at 765 nm. The results are expressed as the mass of total phenol contained in the 100 g sample (fresh weight).

Next, 20 mL of 80% methanol and 1 g of sample were homogenized using liquid nitrogen. After 30 min of ultrasonic extraction, the homogenate was centrifuged at 4 °C, 12,000× *g*, for 20 min. Next, 1 mL of the extract solution was added to 4 mL of 70% ethanol and shaken well, and 0.3 mL of 5% sodium nitrite was added later and rested for 5 min. After that, 0.4 mL of 10% aluminum nitrate was added to the mixture and rested for 6 min. Finally, 4 mL of 1 mol/L NaOH was added and shaken well, stood for 15 min, and then the absorbance was measured at 510 nm. The results are expressed as the mass of flavonoids in a 100 g sample (fresh weight).

Weight loss (%) was calculated using the formula (%) = [(*M*0 − *M*1)/*M*0] × 100 (3)

where *M*0 and *M*1 represent the initial and final weight of each fruit during storage, respectively.

An infrared CO_2_ analyzer was used to measure the respiratory rate (CEA-700, China). Eight fruits were fixed for each treatment, placed in the drier and CO_2_ analyzer simultaneously, and sealed; readings were taken every 20 min three times. The results are expressed in milligrams per kilogram hour of CO_2_.

### 2.7. Statistical Analysis

Microsoft Excel (V. 2019, Washington, USA) was used to calculate the measured values, SPSS (V.26.0, New York, USA)was used to analyze the results, multiple comparisons were made using the ANOVA and Duncan multi-range test (*p* ≤ 0.05), and OriginPro (V. 2021b, Northampton, MA, USA) was used to plot the chart. Principal component analysis (PCA) was performed using the dimensionality reduction factor in SPSS 26.0. Different lowercase letters for the same storage period represent statistically significant differences. Data are presented as mean ± standard error.

## 3. Results

### 3.1. Inhibitory Effect of MT In Vitro

MT exhibited a concentration-dependent inhibitory impact on the mycelial development of *B. dothidea*, as demonstrated in Table 1. The colony diameter after treatment from 0.1 to 1.0 mmol/L MT ranged from 56.17 to 39.17 mm, with 1.0 mmol/L MT producing an inhibition rate of 54% compared with that of the control. The results demonstrated that MT could inhibit *B. dothidea* from growing mycelia.

### 3.2. Effect of Preharvest MT Treatment on Disease Resistance of Kiwifruit

**Figure 1:**
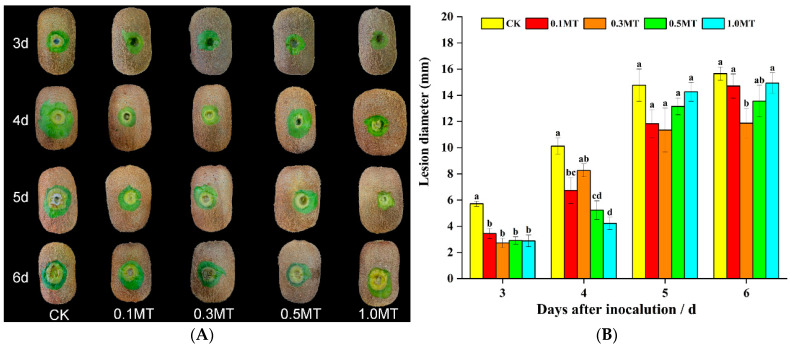
Effect of preharvest melatonin treatment on disease resistance of postharvest kiwifruits. (**A**) Fruit disease phenotype; (**B**) lesion diameter. Values indicate the means of the replicates; error bars indicate the standard deviations of the mean (n = 3). Different lowercase letters indicate significant differences between treatments (*p* < 0.05), while identical letters indicate no significant differences between treatments (*p* > 0.05).

The control group’s lesion sizes were larger than those on the treated kiwifruit fruits treated with different concentrations of MT, as shown in Figure 1A. Figure 1B demonstrates that at 3 d after inoculation, the lesion diameters of the 0.1, 0.3, 0.5, and 1.0 mmol/L MT treatment groups were39.75%, 52.36%, 49.03%, and 49.56% lower than that of the control group, respectively (*p* < 0.05). At 6 d, the lesion diameter of the MT group was lower than that of the control group by 6.06%, 24.20% (*p* < 0.05), 15.96%, and 4.66%, respectively. On day 6, the preharvest 0.3 mmol/L MT treatment group’s lesion diameter was the smallest. However, the lesion diameters of the other MT treatment groups were not significantly different from that of the control group. The various concentrations of MT exhibited a specific control impact on the soft rot on the fruit, as indicated in Table 2. After 18 d of storage, 72.22% of the fruits in the control group were significantly affected by soft rot compared with a much smaller percentage in the other MT groups (*p* < 0.05). The lowest rate among the MT-treated groups affected by soft rot was found for the 0.1 mmol/L MT treatment group. In conclusion, preharvest MT spraying of kiwifruit increased their resistance to soft rot after harvest, with the 0.1–0.3 mmol/L MT treatment producing the best results.

### 3.3. Effect of Preharvest MT Treatment on Appearance Quality of Kiwifruit

The effects of various MT concentrations used for preharvest spraying on the kiwifruit’s morphology and quality are shown in Table 3. The results showed that 0.1, 0.3, and 1.0 mmol/L MT (*p* < 0.05) significantly increased the longitudinal diameter, individual fruit weight, and volume of kiwifruit. However, there was no significant difference in the fruit shape index (*p* > 0.05). In addition, MT treatment significantly enhanced fruit appearance quality, with 1.0 mmol/L MT being the most significant (*p* < 0.05).

### 3.4. Effects of Preharvest MT Treatment on the Storability of Kiwifruit

**Figure 2:**
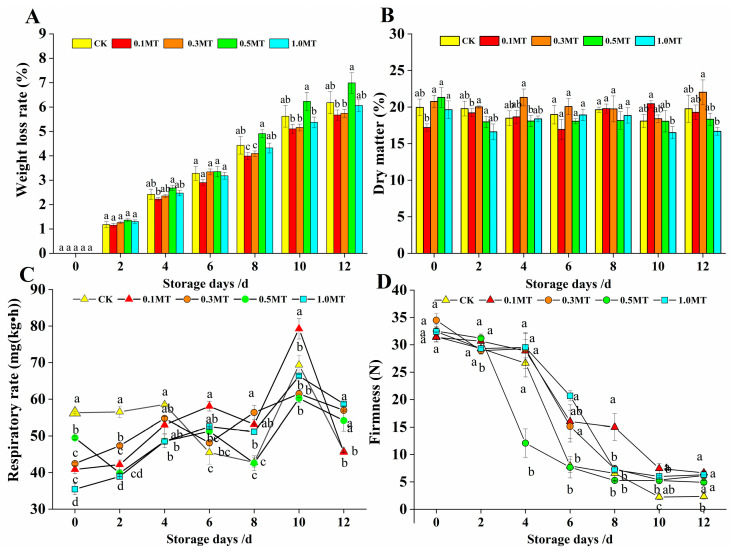
Effect of preharvest melatonin treatment on postharvest kiwifruit weight loss rate (**A**), dry matter (**B**), respiratory rate (**C**), and firmness (**D**). Values indicate the means of the replicates; error bars indicate the standard deviations of the mean (n = 3). Different lowercase letters indicate significant differences between treatments (*p* < 0.05), while identical letters indicate no significant differences between treatments (*p* > 0.05).

The rate of kiwifruit weight loss increased throughout storage, as depicted in Figure 2A. The rate of weight loss in the control and the 0.5 MT treatment groups gradually increased after 8 d of storage, and the rate of increase was significantly higher than that in the other MT-treated groups (*p* < 0.05). However, there was no significant difference in the weight loss rate between the MT and control groups (*p* > 0.05). Figure 2B illustrates how the dry matter content of kiwifruit under ambient storage conditions varied between treatments as storage duration increased. The mass fraction of dry matter in the 0.3 mmol/L MT treatment group reached 22.03% after 12 d of storage; however, there was no significant difference between the treatment groups and the control group (*p* > 0.05). This showed that the preharvest MT treatment could reduce fruit water loss and slow down kiwifruit weight loss, but it had little effect on the dry matter content of the fruit postharvest.

At 0–2 d of storage, the respiratory rates of each treatment group were significantly lower than those the control group (*p* < 0.05), as shown in Figure 2C, and the fruit’s respiratory intensity increased as the storage period increased. However, the 0.1 MT treatment reached peak value (79.24 mg CO_2_/(kg·h)) at 10 d, and then the respiratory intensity dropped at the end of storage. This showed that the preharvest spraying of MT on fruit surfaces could successfully inhibit the kiwifruit’s respiratory intensity compared with that of the control during storage.

One indicator for determining the ripeness of the fruit is its firmness [30]. Figure 2D shows that fruit firmness slightly declined as the storage period increased. However, the fruit firmness of the other treatment groups remained relatively high except for that in the 0.5 mmol/L MT and control groups, whose firmness significantly dropped after 4–6 d of storage. The firmness of the 0.1 mmol/L MT group was higher than that of the treatment and control groups at 8 d. The fruit firmness of the kiwifruit in each treatment group was higher than that in the control group at the end of storage (*p* < 0.05). The firmness was 6.67 N after storage, and the impact of 0.1 mmol/L MT on firmness was better. This indicated that preharvest MT treatment could effectively delay fruit softening and prolong the shelf life of kiwifruit.

**Figure 3:**
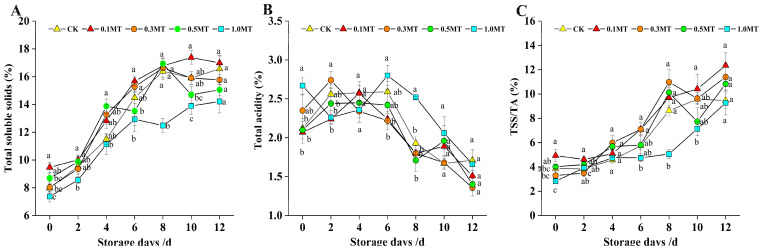
Effect of preharvest melatonin treatment on postharvest kiwifruit total soluble solids (**A**), titratable acidity (**B**), and TSS/TA (**C**). Values indicate the means of the replicates; error bars indicate the standard deviations of the mean (n = 3). Different lowercase letters indicate significant differences between treatments (*p* < 0.05), while identical letters indicate no significant differences between treatments (*p* > 0.05).

One of the parameters used to evaluate kiwifruit flavor is TSS/TA, and TSS increases as the fruit ripens, while TA decreases as the storage period increases [28]. The TSS content of each treatment group was between 7 and 9% at 0 d, whereas that of TA was above 2%, as illustrated in Figure 3A–C. The TSS and TA contents displayed a gradually increasing and decreasing trend with increased storage duration, respectively. The TSS and TA were lower and higher, respectively, in the 1.0 MT treatment group after 8 d of storage than in the other treatment and control groups (*p* < 0.05). However, at 10–12 d of storage, there was no significant difference in their contents between the treatment and control groups (*p* > 0.05). The results indicated that preharvest MT (1.0 mM) treatment might prevent kiwifruit from softening, delay the increase in TSS, lessen the loss of TA during storage, and maintain a healthy ratio of TSS to TA.

### 3.5. Effects of Preharvest MT Treatment on Nutritional Components of Postharvest Kiwifruit

**Figure 4:**
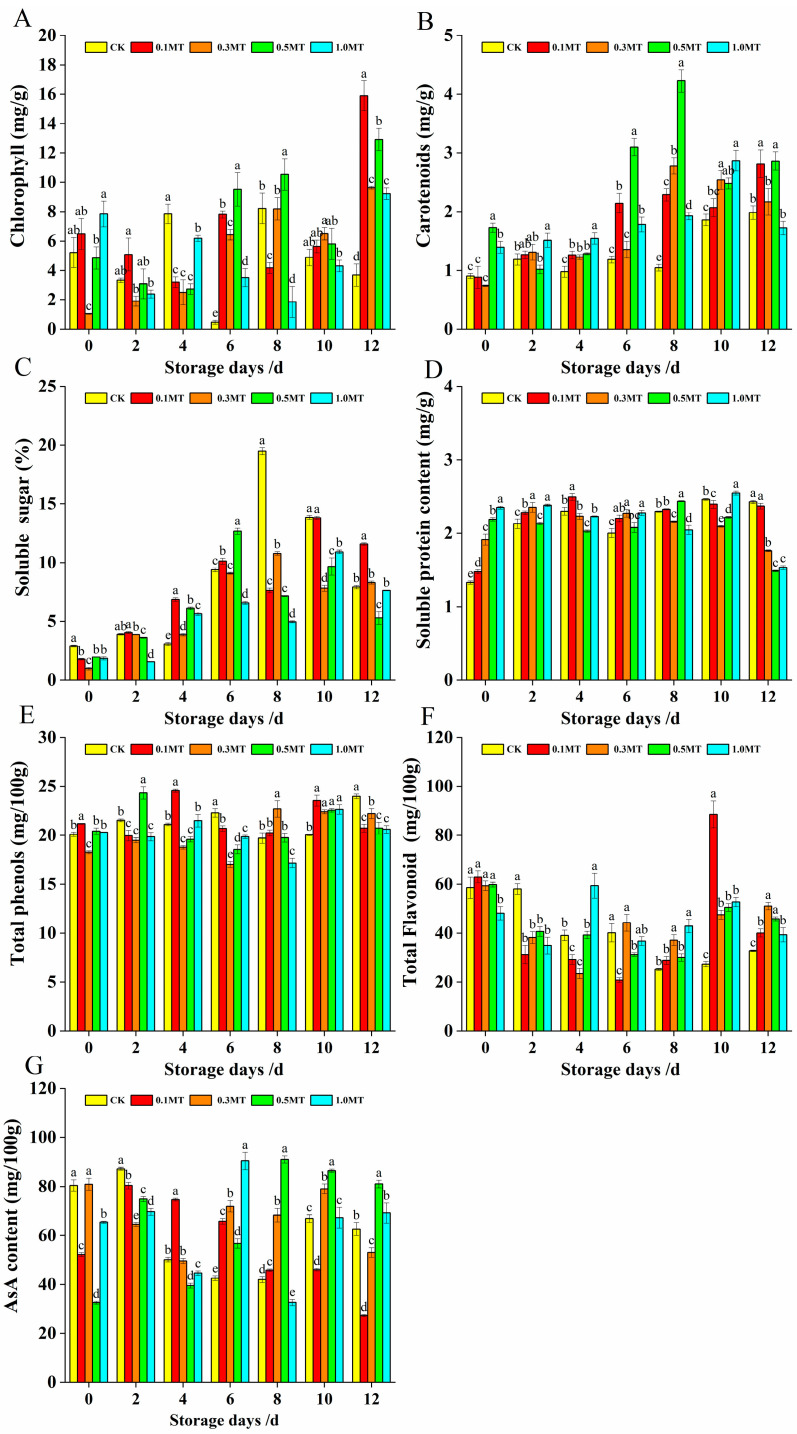
Effect of preharvest melatonin treatment on the nutrient composition of postharvest kiwifruits. (**A**). Chlorophyll (**B**). Carotenoids, (**C**). Soluble sugar (**D**). Soluble protein (**E**). Total phenols (**F**). Total flavonoids, (**G**). Ascorbic acid. Values indicate the means of the replicates; error bars indicate the standard deviations of the mean (n = 3). Different lowercase letters indicate significant differences between treatments (*p* < 0.05), while identical letters indicate no significant differences between treatments (*p* > 0.05).

Figure 4A,B show how the amounts of chlorophyll and carotenoids in the kiwifruit steadily rose as the fruit softened and matured. The content of carotenoids in the 0.5 mmol/L MT group peaked at 8 d of storage (4.227 mg/g), which was significantly higher than that in the other treatment and control groups (*p* < 0.05). In contrast, the effect of 0.1 mmol/L MT and 0.3 mmol/L MT on the carotenoid content at the end of storage was better than that in the other treatments and control groups. The chlorophyll content of the control group was only 3.684 mg/g at 12 d, which was lower than that in the MT treatment groups (*p* < 0.05). These results demonstrated that preharvest MT application by spraying boosts chlorophyll and carotenoids during storage and supports the preservation of fruit quality.

Figure 4C,D show that he soluble sugar in kiwifruit gradually increased as the storage duration increased, although the soluble protein content displayed divergent tendencies. The content in all other treatment groups exhibited a fluctuating reduction, except for that in the control group and 0.1 mmol/L MT group. At 0 d of storage, the control group’s contents of soluble sugar and soluble protein were higher and lower than those of the treatment groups, respectively (*p* < 0.05). At 8 d of storage, the control group’s soluble sugar content was 19.48%, being significantly higher than that in the MT treatment groups (*p* < 0.05). Then, at 12 d of storage, the effect was stronger with 0.1 MT. The results demonstrated that preharvest MT spraying inhibits the growth of the kiwifruit’s soluble sugar mass fraction in the later stages of storage, inhibits the loss of soluble protein, and prevents the loss of fruit quality during kiwifruit fruit storage. The above findings significantly contribute to the knowledge of fruit storage and preservation.

The changes in the total phenolic and flavonoid contents of the kiwifruit are depicted in Figure 4E,F. At 10 d of storage, the contents of total phenols and flavonoids in each treatment group were significantly higher than those in the control group (*p* < 0.05). For example, the content of total phenols in the 0.1 mmol/L MT group was 23.55 mg/100 g and the content of flavonoids was 88.54 mg/100 g at 10 d of storage. The results also revealed that the contents of total phenols and flavonoids in kiwifruit demonstrated a fluctuation pattern during the 12 d storage period.

According to Figure 4G, each treatment group’s AsA content experienced a declining trend during storage. However, the AsA content of the kiwifruit in each treatment group was significantly higher than that in the control group after 6 d of storage (*p* < 0.05). At the end of storage, the AsA content in the 0.5 mmol/L MT group was 81.12 mg/100 g, a better result than that achieved in the other MT groups and control group.

### 3.6. Correlation Analysis of Preharvest MT Treatment on Kiwifruit Storage Quality

**Figure 5:**
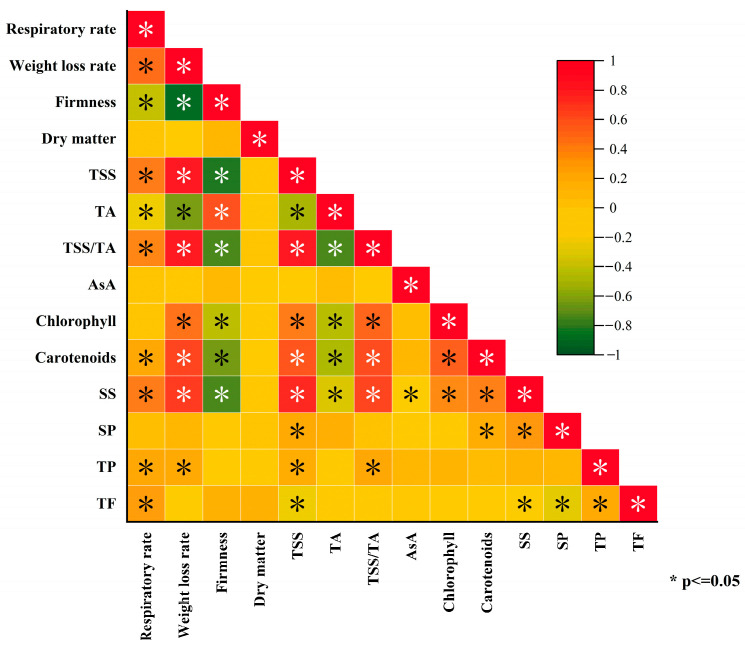
Correlation analysis of quality of kiwifruit under room-temperature storage. Note: * represents a significant difference between *p* ≤ 0.05.

According to Figure 5, the postharvest kiwifruit’s maturity and aging indicators, such as respiratory and weight loss rates, were positively correlated with TSS, carotenoids, SS, TP, TF, and other physical and chemical properties and nutritional components, and negatively correlated with firmness and TA. Firmness and TA, however, were negatively correlated with SS, carotenoids, chlorophyll, and TSS, while TSS and TSS/TA were positively correlated with carotenoids, chlorophyll, SS, and TP.

### 3.7. Principal Component Analysis of Preharvest MT Treatment on Kiwifruit Storage Quality

The principal component analysis (PCA) method was used to thoroughly evaluate the 15 quality indicators of *Guichang* fruit, and the dimensionality reduction of these 15 indicators was obtained. The PCA evaluation was performed to accurately assess the effect of preharvest spraying of different concentrations of MT on postharvest kiwifruit quality. The loading matrix for the first four primary components is displayed in Table 4. The principal components 1, 2, and 3 with eigenvalues greater than 1 were taken from the fruit quality index of *Guichang* kiwifruit, and their corresponding values were 6.210, 1.658, 1.372, and 1.278, respectively. The comprehensive evaluation score results from multiplying each principal component’s score were obtained as the factor score in SPSS 26.0 software multiplied by the arithmetic square root of the eigenvalue by its corresponding contribution rate. The impact of various melatonin concentrations on the postharvest kiwifruit fruit quality was determined. The following was the formula used to determine the thorough evaluation score:

*Y =* 0.41398*Y*_1_
*+* 0.11053*Y*_2_
*+* 0.09144*Y*_3_
*+* 0.08518*Y*_4_ (4)

In Formula (4), *Y* represents the comprehensive evaluation score; *Y*_1_, *Y*_2_, *Y*_3_, and *Y*_4_ represent the score of the principal components 1, 2, 3, and 4, respectively. Table 5 displays the total kiwifruit quality score for each treatment group. Table 5 shows that, in descending order, the total scores of the various melatonin concentrations on the quality of postharvest kiwifruit were 0.3 MT, 0.1 MT, CK, 0.5 MT, and 1.0 mmol/L MT.

## 4. Discussion

A typical postharvest storage disease of kiwifruit is soft rot [31]. Prior research has demonstrated that MT has various inhibitory effects on fungal infections [32,33,34]. However, the primary source of the developmental abnormalities in mycelia is the breakdown of organelles produced by high MT concentration [35]. This study revealed that MT inhibited *B. dothidea* at various concentrations, with the 1.0 mmol/L MT treatment group outperforming the other treatment groups. Most research has shown that postharvest MT application can effectively create fruit resistance to disease invasion [36,37,38]. Using 0.1–0.3 mmol/L MT may promote the synthesis of endogenous MT in kiwifruit, thereby inducing disease resistance signal transmission to prevent pathogen infection. Additionally, inoculation experiments showed that spraying MT during kiwifruit growth and development stage effectively prevented postharvest *B. dothidea* expansion and controlled the incidence of soft rot. This result is consistent with the results reported by Li et al. [24].

The kiwifruit morphology plays a crucial role in luring customers and is one of the critical markers of postharvest grading and selection [38]. According to earlier research, preharvest exogenous melatonin treatment boosted fruit yields and weights in grapes, pomegranates, and sweet cherries [39,40,41]. In this study, preharvest melatonin spraying significantly maintained the weight and volume of a single kiwifruit fruit. It played a beneficial function in the fruit’s growth and development, comparable to the finding for pears [42]. While the weight loss rate, respiratory rate, and firmness are the most logical indicators for determining the ripening and senescence of postharvest fruits, the change in internal quality during storage dictates the storage performance and shelf life of kiwifruit [43,44]. Our findings correspond with those obtained on date palms, pomegranates, apricots, and cherries [35,39,40,41]. Furthermore, the findings suggest that preharvest MT treatment could reduce the respiratory rate of kiwifruit during early storage and retain its firmness at the end of storage [40,45,46,47]. These findings show that MT slows the softening and senescence of kiwifruit by lowering the respiration rate, hence reducing the water content of kiwifruit during storage.

Through photosynthesis, plants produce sucrose and starch, while compounds such as phenolics and ketones are produced when organic materials such as sugars undergo secondary metabolism [40,41,44]. Fruits’ phenolics and ketones directly impact their color, luster, flavor, storage, and processing properties. Although astringency, sourness, sweetness, and other flavors are intimately related to phenolics and ketones [48], the pulp’s hue is closely tied to the efficacy of an antioxidant [49]. A good source of natural antioxidants is chlorophyll. The findings of this study showed that preharvest 1.0 mmol/L MT treatment significantly reduced kiwifruit TSS and SS content increases while maintaining high TA and SP contents. At the same time, MT treatment increased the contents of TP, TF, chlorophyll, carotenoids, and AsA. The correlation results confirmed that the sugar content and phenolic pigments in kiwifruit are positively correlated. These findings suggest that MT positively affects kiwifruit disease inhibition.

Principal component analysis (PCA) is a multivariate statistical technique that reduces the dimensionality of data by generalizing a large number of linked indicators [50]. Principal component analysis has been widely used to analyze a variety of horticultural crops, including guava, peach and blueberry [51,52,53]. The effect of melatonin treatment on postharvest quality and oxidative stress markers of litchi fruit during cold storage was determined using PCA as well as correlation analysis, showing that melatonin treatment effectively delayed the fruit senescence by enhancing the antioxidant enzyme activities and modulating peel browning [54]. Paulina et al. [55] identified the most important variables in the relationship between 20 selected peach varieties based on PCA model and identified the most attractive varieties.

The PCA revealed that four principal components with eigenvalues greater than one were retrieved in this experiment, with a total contribution of 70.11%. Solid acid ratio, hardness, soluble solids, titratable acids, carotenoids, soluble sugars, and chlorophyll could be employed as primary markers for kiwifruit quality evaluation. Using principal component analysis, we determined that 0.3 mmol/L MT had the strongest impact on enhancing kiwifruit disease resistance and preserving fruit quality. The effect of nutrient application in the field on improving the agronomic qualities of horticultural products has been acknowledged to some extent. However, many unanswered questions remain regarding the postharvest biological characteristics of fruits and vegetables. In particular, there is a shortage of research on the biological stress experienced by fruits and vegetables. This research studied the control effects produced by melatonin from the standpoint of fruit–pathogen–environment interaction, and the findings offer support for the more effective use of melatonin in production practice. Additionally, it will be very important to further study how exogenous melatonin induces fruit resistance by influencing endogenous melatonin synthesis.

## 5. Conclusions

The results of this investigation demonstrate that preharvest melatonin spraying effectively prevents soft rot. In addition, MT inhibits the mycelial growth of *B. dothidea,* and preharvest MT spraying improves the postharvest resistance of kiwifruit to soft rot. The best effects were observed at 0.3 mM, and MT spraying raised kiwifruit’s fruit weight, maintained fruit firmness, reduced respiration rate, and improved quality. This work offers a fresh approach to kiwifruit quality enhancement and postharvest disease management.

## Figures and Tables

**Table 1:** In vitro inhibition of *B. dothidea* by different concentrations of MT.

Treatment	Colony Diameter (mm)	Inhibitory Rate (%)
Control	78.33 ± 2.49 ^a^	-
0.1 MT	56.17 ± 4.76 ^b^	30.65 ± 6.59 ^c^
0.3 MT	54.67 ± 5.18 ^bc^	32.72 ± 6.59 ^bc^
0.5 MT	41.67 ± 0.33 ^cd^	50.69 ± 1.74 ^ab^
1.0 MT	39.17 ± 5.99 ^d^	54.19 ± 8.28 ^a^

Values indicate the mean ± SD, n = 3. Upper case letters indicate significant differences at 5% level (*p* < 0.05). Different lowercase letters indicate significant differences between treatments (*p* < 0.05), while identical letters indicate no significant differences between treatments (*p* > 0.05).

**Table 2:** Control efficiency of MT against soft rot of kiwifruits.

Treatment	Incidence (%)	Disease Severity Value
Control	72.22 ± 0.04 ^c^	53.06 ± 0.04 ^d^
0.1 MT	41.25 ± 0.02 ^a^	31.88 ± 0.02 ^a^
0.3 MT	56.88 ± 0.03 ^b^	40.44 ± 0.01 ^c^
0.5 MT	55.56 ± 0.04 ^b^	37.88 ± 0.01 ^b^
1.0 MT	56.25 ± 0.03 ^b^	40.64 ± 0.01 ^c^

Values indicate the mean ± SD, n = 3. Upper case letters indicate significant differences at 5% level (*p* < 0.05). Different lowercase letters indicate significant differences between treatments (*p* < 0.05), while identical letters indicate no significant differences between treatments (*p* > 0.05).

**Table 3:** Effect of MT on the development of kiwifruits.

Treatment	Longitudinal Diameter (mm)	Transverse Diameter (mm)	Lateral Diameter (mm)	Single Fruit Weight (g)	Single Fruit Volume (cm^3^)	Fruit Shape Index
Control	65.00 ± 1.41 ^c^	35.55 ± 0.16 ^b^	41.18 ± 0.27 ^b^	61.52 ± 2.08 ^b^	56.06 ± 1.52 ^c^	1.83 ± 0.03 ^a^
0.1 MT	68.30 ± 0.87 ^ab^	36.24 ± 0.06 ^ab^	42.40 ± 0.34 ^a^	68.61 ± 0.36 ^a^	61.28 ± 0.53 ^ab^	1.90 ± 0.01 ^a^
0.3 MT	68.31 ± 0.57 ^ab^	36.65 ± 0.48 ^a^	42.29 ± 0.27 ^ab^	68.23 ± 0.65 ^a^	60.04 ± 0.49 ^b^	1.86 ± 0.05 ^a^
0.5 MT	66.02 ± 0.50 ^bc^	35.42 ± 0.33 ^b^	42.90 ± 0.55 ^a^	63.64 ± 0.53 ^b^	58.83 ± 0.47 ^b^	1.86 ± 0.00 ^a^
1.0 MT	69.80 ± 0.31 ^a^	36.39 ± 0.22 ^ab^	42.82 ± 1.39 ^a^	70.56 ± 0.97 ^a^	62.57 ± 0.62 ^a^	1.92 ± 0.02 ^a^

Values indicate the mean ± SD, n = 3. Uppercase letters indicate significant differences at 5% level (*p* < 0.05). Different lowercase letters indicate significant differences between treatments (*p* < 0.05), while identical letters indicate no significant differences between treatments (*p* > 0.05).

**Table 4:** Rotated component matrix of principal component analysis.

Indicator	*Actinidia* *deliciosa* Guichang			
	Principal Component 1	Principal Component 2	Principal Component 3	Principal Component 4
Solid acid ratio	0.905	0.028	0.142	−0.072
Weight loss rate	0.898	0.173	0.202	0.044
Firmness	−0.891	−0.182	−0.12	0.035
Total soluble solids	0.836	0.353	0.138	−0.097
Titratable acidity	−0.783	0.332	−0.042	0.101
Disease incidence	0.774	−0.154	0.279	−0.067
Carotenoids	0.733	0.109	−0.04	0.278
Soluble sugar	0.67	0.486	0.177	−0.173
Chlorophyll	0.655	−0.159	−0.202	0.244
Soluble protein	−0.024	0.835	0.074	0.002
Respiratory rate	0.34	0.088	0.721	−0.112
Total phenols	0.102	0.076	0.687	0.292
Total flavonoids	−0.169	−0.561	0.596	−0.202
Ascorbic acid	−0.084	−0.134	0.08	0.765
Dry matter	−0.058	−0.132	0.014	−0.647
Eigenvalue	6.21	1.658	1.372	1.278
contribution ratio	41.398	52.451	61.595	70.113

**Table 5:** Comprehensive evaluation results of different melatonin concentrations on postharvest kiwifruits quality.

Treatment	Score of Each Principal Component				Comprehensive Score	Ranking
	*Y*_1_	*Y*_2_	*Y*_3_	*Y*_4_		
Control	2.813	−0.513	−0.357	0.273	1.100	3
0.1 MT	3.877	−0.270	0.293	−1.757	1.453	2
0.3 MT	3.400	2.407	0.053	−1.190	1.577	1
0.5 MT	2.817	1.513	−2.497	−0.403	1.073	4
1.0 MT	2.580	1.440	−1.837	−0.187	1.040	5

## Data Availability

The data used to support the findings of this study can be made available by the corresponding author upon request.
